# Transgender Competent Provider: Identifying Transgender Health Needs, Health Disparities, and Health Coverage

**Published:** 2018-02-28

**Authors:** Sarah Houssayni, Kari Nilsen

**Affiliations:** Department of Family and Community Medicine, University of Kansas School of Medicine-Wichita, Wichita, Kansas

**Keywords:** transgender, gender identity, sexual minorities, gender confirmation procedures, culturally competent care

## Introduction

Transgender individuals have gender identity, gender expression, or behavior that differs from their biological sex assigned at birth.^1^ It is estimated that 0.3% of US adults identify as transgender, which is nearly 1 million people.^2^ While much is unknown in the field of gender incongruence, “a growing and persuasive body of evidence suggests that biological factors have a substantial role in predisposing some people towards gender incongruence”.^2^

The earliest recognition of gender nonconformity in Western medicine was in the 1920s and was labeled as a mental pathology.^3^ Gender Identity Disorder (GID) became a diagnosis in the American Psychiatric Association’s third edition of the *Diagnostic Manual of Mental Disorders* (DSM) in the 1980s. GID was a mental pathology until the DSM-5^4^ was released in 2012, when GID was dropped as a diagnostic condition. Now, the DSM-5 has only an overarching diagnosis of gender dysphoria. This newly defined diagnosis does not pathologize being transgender, instead it can occur when an individual who is transgender has distress related to the incongruence between his/her experienced and expressed gender. Psychological treatment targets coexisting emotional and mental morbidities. Thus, the trend has shifted from attempting to treat gender nonconforming individuals to being more accepting of them.^2^

To that end, this overview addresses what it takes to be a competent transgender provider, including knowing health needs, health disparities, and health coverage. Next, medical biases toward transgender patients are examined and current hormone treatment guidelines are outlined for both transgender men and women. Also, treatment options for transgender children and adolescents, along with aging patients, are discussed. The discussion concludes with trends, movements, and expectations for medical curricula.

## Health Needs

Besides being a social minority, transgender individuals are a health minority who face myriad challenges as outlined in the National Transgender Discrimination Survey.^5^ The challenges outlined in the survey include facing serious acts of discrimination, such as loss of job, eviction, bullying, physical or sexual assault, denial of medical services, and incarceration. Barriers faced by transgender people in society and in the medical system make this group more likely to experience mental illness, discrimination, and poor health outcomes.^3^ For example, prevalence of psychiatric diagnoses appears to be high, especially for lifetime depressive episode estimated at 35.4%, and suicidality for transgender women at 20.2%.^6^

Along with traditional health care, transgender individuals have unique needs related to gender transitioning, specifically mental health, hormonal treatment and side effects, and adequate referral to surgery, which can “create an undesired and unavoidable dependency on the medical system.”^3^ Procedures may include facial feminization/masculinization surgery, voice, breast, or chest surgery, hysterectomy, and genital reconstruction.^7^ Patients who experience obstacles in obtaining health services may experience short- and long-term negative health consequences. Of particular concern, one in four transgender women self-prescribe cross-sex hormones and obtain them illegally (most commonly via the internet), a practice that may be hazardous to health.^8^ This practice rarely was observed for transgender men.

## Health Disparities

Healthy People 2020 outlines the need to research factors contributing to health disparities in the Lesbian, Gay, Bisexual, and Transgender (LGBT) community.^9^ A survey was conducted in 2008 by the National Center for Transgender Equality and the National Gay and Lesbian Task Force to understand these health disparities.^5^ Participants included 7,500 individuals. Results showed that 19% of respondents reported being denied health care based on their gender identity, and 28% reported verbal harassment in a medical setting. A third of respondents avoided preventive care, while 28% reported postponing any medical care due to discrimination and disrespect. Findings showed a higher than average likelihood of human immunodeficiency virus (HIV) among transgender patients. Respondents also were more likely to depend on drugs and alcohol to cope with negative experiences, while a higher likelihood of lifetime suicide attempts was revealed: 41% compared with 1.6% in the general population. This percentage was for both male and female respondents. Racial minorities who are transgender appeared to experience the highest risk for negative health outcomes and risk-taking behaviors.

Almost ten years later, the National Transgender Discrimination Survey was conducted with 6,450 transgender and gender non-conforming participants.^5^ Experiences of discrimination where equal treatment was denied was reported by 24% of participants in doctor’s offices or hospitals, 13% in emergency rooms, 11% in mental health clinics, and 5% for ambulance or emergency medical services. Participants also reported being harassed or disrespected at even higher rates for these same locations.

Results of these studies, among others, led the Association of American Medical Colleges (AAMC) to conclude that transgender health needs are not addressed adequately, and education for medical providers and staff about transgender health and cultural competencies is necessary. Thus, they convened an advisory committee in 2012 to develop a set of competencies for undergraduate medical education.^10^

## Health Coverage

In 2011, the Veterans Health Administration in the US Department of Veterans Affairs (VA) issued directives to support covering medical care and hormonal treatment for transgender patients. However, it denied sex reassignment surgery (SRS) based on the argument it comprises genital alteration, which is excluded from veterans’ medical benefits packages. Statements from the current Directive 2013-003, which expires on Feb. 28, 2018, are:

1. Establishes policy regarding the respectful delivery of health care to transgender and intersex veterans.
2. Provides health care for transgender patients, including those who present at various points on their transition from one gender to the next.
3. Medically necessary care is provided to enrolled or otherwise eligible intersex and transgender veterans, including hormonal therapy, mental health care, preoperative evaluation, and medically necessary post-operative and long-term care following sex reassignment surgery.

But, the directive explicitly stated that SRS cannot be performed or funded by the VA.^11^

Many private insurance companies have included hormonal treatment, except Medicaid, unless a letter of medical necessity and the terminology “hormonal replacement” are used.^12^ Coverage is expected to change in favor of hormonal treatment, given a 2012, US Department of Health and Human Services (HHS) statement clarifying that the ban on sex discrimination in section 1557 (nondiscrimination) includes discrimination based on gender identity.^13^ Further, on May 30, 2014, the US HHS Departmental Appeals Board determined the National Coverage Determination denying coverage for all transsexual surgery was not valid. As a result, Medicare Administrative Contractors determine coverage on a case-by-case basis.^12^

Treatment appears to alleviate gender dysphoria and have positive effects on quality of life.^14^,^15^ However, a scoping review observed that many published articles on the sexual health of transgender men come from limited data with potentially biased samples, citing controversial recruitment practices and high dropout rates, which may inflate the positive effects of therapy.^16^ In addition, a systematic review stated a need for new self-assessment tools to evaluate functional, psycho-relational, cosmetic, and quality of life of patients who undergo transsexual surgery.^17^

Despite increased efforts to support transgender health, linking medical care to transition care remains the most common cause of coverage denial. Perhaps insurers will agree to enhance coverage after more research is conducted (with less biased samples and new evaluation tools) that verify the benefit of SRS.

## Medical Biases toward Transgender Patients

Transgender individuals experience many barriers to healthcare.^18^ Among these were: 1) fear of being seen as different (with associated stigma and violence), 2) lack of access to caring and competent professionals, 3) difficulty in identifying sources of information about gender dysphoria and hormone therapies, and 4) inadequate access to safe prescribing and monitoring of hormone therapy. One of the most commonly reported barriers to healthcare identified by transgender individuals is their ability to find a knowledgeable provider.^19^ Fifty percent of transgender patients reported having to educate their providers about transgender health in a 2014 survey.^20^

Medical school and residency curricula are lacking in transgender health.^18^ Culturally competent language and sensitive approaches to transgender individuals are lacking from most medical training.^21^ In 2014, only one third of US medical schools had any teaching or training in transgender health, and the ones that did may not be adequate to ensure enough knowledge to make providers comfortable to treat transgender patients.^22^ One theory for the discrepancy between patient need and medical availability is the belief among medical providers that transgender patients suffer from a psychological disorder. Although the DSM-5^4^ has changed the terminology around transgenderism, it has kept it as a diagnosable condition that can occur when an individual who is transgender has distress related to the incongruence between his/her experienced and expressed gender.^21^

A growing body of research indicates that gender identity can be independent of chromosomal findings.^15^ Transgender patients’ brains correlated with physical manifestations of gender identity. Male transgender individuals’ brains showed white matter microstructure more similar to cisgender males than cisgender females. Female transgender individuals without hormonal treatment had brain characteristics similar to cisgender females (e.g., size of the bed nucleus of the stria terminalis in the hypothalamus within the female range). In summary, individuals with a certain gender identity have anatomic brain findings consistent with their preferred biological sex, despite the sex assigned to them at birth.

## Standards of Care

Standards of care (SOC) have been developed by the World Professional Association for Transgender Health (WPATH).^23^ WPATH standards include primary care, gynecologic and urologic care, reproductive options, voice and communication therapy, mental health services, along with hormonal and surgical treatments. A WPATH Mobile App is available at Google Play and iOS at the iTunes Store. The guideline from the Center of Excellence for Transgender Health complements WPATH, and is designed for implementation in every day evidence-based primary care settings.

### Hormonal Regimen for Transgender Men

Transgender males are assigned female sex at birth, but self-identify as male.^15^ Their hormonal treatment consists of administering testosterone to achieve maximum virilization. The levels are increased by pharmacological administration (intramuscular or patch) until the measured testosterone levels are within the normal male range (300 – 1000 ng/dl). Testosterone can be administered orally, however, that preparation is not available in the US. The most commonly used form in the US is intramuscular with doses of 50 – 200 mg weekly. Patients can go to bi-weekly dosing and administer higher doses (100 – 200 mg intramuscular) themselves, but the levels of testosterone fluctuate more with a bi-weekly regimen. Transdermal preparations (patch of 2.5 – 7.5 mg daily), testosterone 1% gel (2.5 – 20g/day), or intramuscular testosterone work, but may cause skin irritation and virilizing side effects to family members contaminated with the gel or cream if patients are not careful.

Transgender men must be followed initially every three months to check for virilizing and side effects.^15^ The main effects observed are loss of periods (amenorrhea), increased facial and body hair, increased muscle mass with changed fat distribution to male pattern, increased acne, and increased libido. Deepening of the voice, enlarged clitoris (clitoromegaly), and male pattern hair loss occur to varying extent in different individuals and occur over the first year. Transgender men starting hormonal treatment after the age of 40 may see less virilizing effects.

### Follow-up and Side Effects

Transgender males require follow-up every three months over the first year, then every six months the following year and yearly, if lab tests are within acceptable limits.^15^ Testosterone levels are obtained at every visit; the dose can be modified to achieve a level in the male range (300 – 1000 ng/dl). Hematocrit and lipid levels are monitored. A bone scan is obtained at the beginning of treatment if patients are at risk of osteoporosis or not achieving adequate levels, otherwise the bone scan screening starts at age 60. Transgender males with breast tissue and cervixes need appropriate screening.

Transgender males on testosterone will have reduced fertility and are less likely to get pregnant.^15^ Those effects are permanent even with stopping testosterone. Pregnancy is possible and that should be explained to transgender males who engage in sexual activity with partners with sperm (fertile non-transgender men or fertile transgender women). Transgender men are also at the risk of contracting sexually transmitted infections with unprotected sex.

### Hormonal Regimen for Transgender Women

Transgender women born with male biologic sex require a blockage of testosterone action (anti-androgen) besides increasing estrogen levels.^15^ Treating transgender women aims at decreasing testosterone to the female range (30 – 100 ng/dl) and obtaining an estrogen level not exceeding the physiological female range (< 200 pg/ml). Treating with anti-androgens, such as spironolactone, allows for lower doses of estrogen. Spironolactone is used orally in a dose of 100 – 200 mg daily, but may be used up to 400 mg if needed. Spironolactone is a mild potassium sparing diuretic, hence the need to monitor levels of potassium. Estrogen is administered orally (2.5 – 7.5 mg of estrogen or 17-beta estradiol at 2 – 6 mg) daily or intramuscularly (estradiol valerate 2 – 10 mg once a week or 5 – 20 mg every two weeks). Patches can be used (estradiol patch 0.1 – 0.4 mg, two times a week) if the transgender woman is at increased risk of thromboembolic disease.

### Changes

Body hair decreased, skin was less oily, muscle mass decreased with redistribution of body fat in a female pattern, spontaneous erections decreased, and libido and breast development decreased within three to six months. Breasts reached peak size typically after two years of hormonal treatment.^15^

### Monitoring and Side Effects

Transgender women on hormonal treatment must be monitored with testosterone levels (suppressed to 30 – 100 ng/dl) and estradiol levels increased but remaining below 200 pg/ml.^15^ They also must be monitored for potassium levels due to the risk of having high levels with testosterone-blocking spironolactone. The levels of prolactin and triglycerides need checked with labs, since estrogen treatment may cause hyperlipidemia.

## Transgender Children and Adolescents

Many pre-pubertal patients with varying features of gender dysphoria will become transgender teenagers.^18^ Most transgender teenagers experience gender identity conflicts as children. Children who identify with a gender different from one assigned at birth often become gay and lesbian adults and do not become transgender adolescents. The child’s reaction to beginning puberty is often diagnostic. The guidelines recommend allowing puberty to start without medical intervention, however, if not desired, puberty can be blocked early on (at Tanner stages 2 and 3), and hormonal treatment started when the patient is deemed ready (age 18 or 16 with parental consent). Puberty suppression is done for patients who are non-transgender with precocious puberty to avoid permanent short stature. Some medical professionals are familiar with puberty blockage, however, not for transgender patients. The trend for transgender health awareness and education targets this gap area to keep transgender teenagers from going through the “wrong puberty”.

Puberty suppression for teenage transgender individuals reduces the risk of emotional and behavioral problems and increases functioning.^18^ A study of a simple one-hour transgender curriculum at a Boston University Medical School showed improved student willingness to treat transgender patients, and increased perceived knowledge and comfort level.^19^

## Assessment of a Teenager with Gender Dysphoria

Whereas the majority of preadolescent individuals seeking medical attention for gender dysphoria are born with male gender and identify as girls, the ratio in adolescence is close to a ratio of 1:1.^24^ One explanation for transgender girls attracting parental and medical attention is that society is more accepting and less alarmed about female-born individuals who dress and act in a masculine fashion. “Tomboy” is a term used for those individuals with no equivalent for their male-born counterparts. Regardless of when gender dysphoria manifests, the consistency, persistence, and insistence of a teenager with an identified gender that is not the one assigned at birth should be taken seriously. Suicidal ideation, depression, and self-medication increase remarkably at this age.^21^ Ideally, a multidisciplinary team with a psychologist, social worker, and physician are available for those patients and families. It often is not the case and many teenage transgender patients avoid medical care and some will attempt conforming to their birth gender, typically without success, and many secondary emotional consequences.

A medical provider lacking the sensitivity and cultural competence to engage a transgender patient, especially a teenager, will miss signs of gender dysphoria and potentially cause harm by saying gender stereotypical things that alienate the patient further.^22^ Medical providers interacting with teenagers should establish a safe space and inform patients about confidentiality in all matters, excluding homicidal and suicidal intentions. Once patients feel safe to discuss private matters, they likely will be willing to talk about any gender struggles they may be experiencing.

Competent clinicians who provide care for teenage patients are expected to screen for emotional and social stressors as part of an interview during their yearly well check. If an adolescent discloses concerns about gender, the clinician should be able to screen for gender dysphoria and be prepared to suggest resources to help the patient and their family. A mental health clinician is often the first place to obtain a diagnosis and suggest future steps. Gender dysphoria screening is not recommended in every teenage patient who is not gender conforming, but should be screened for well-being and personal struggles.

Once a clear and consistent gender identity is established and mental health providers, family, and patient agree on treatment regimen, hormonal replacement can start.^18^ Ideally, puberty would have been blocked and minimal secondary sexual characteristics of the birth gender are present. Puberty blockage usually is done with gonadotropin-releasing hormone (GnRH) analogues. Patients must be monitored with bone scans, as height and bone density growth slows during treatment with GnRH analogues. Puberty blockage allows for the need to use fewer hormones to establish the desired secondary sexual characteristics (facial hair, deep voice, and male body habitus in transgender females; breast development and female body habitus in male transgender) and has been correlated with less psychosocial negative outcomes in transgender teens. Progestins are used in the transgender male teenager to avoid menstruation and cyproterone can suppress erections and nocturnal emissions in female transgender teenagers. However, the latter medication can cause breast tissue development and is not recommended without ensuring that the transgender identity is well established for the teenager.

Parental or legal guardian consent is necessary in treatment for transgender teenagers.^17^ Consent is a problem for many teenagers with unsupportive families and has been associated with maladaptive behaviors, such as running away from home, and risky, self-destructive behaviors. Visibly gender non-conforming teenagers are vulnerable to harassment and hate crimes. The options of teenage transgender individuals are limited by their financial dependence on their families and abilities to become financially independent. Homeless transgender teenagers have a challenge in finding housing options, as they often are placed based on the gender of their birth.

## Aging Transgender Guidelines for Screening and Preventative Health

Hormonal replacement has no age limit. However, transgender males who stop testosterone lose their facial and body hair, muscle mass, suffer from decreased libido, and develop hot flashes.^15^ It is not recommended to stop hormones if the patient has had resection of their birth gonads (post-gonadectomy) because both men and women are at risk of bone loss and symptoms similar to post menopause. Osteoporosis has been reported in both transgender men and women with poor hormone regimen. Surgical confirming procedures carry a higher risk for older patients, as do most major surgical procedures, and those risks should be discussed with the surgeon (surgical team) involved with gender confirmation surgery.^25^

Transgender females on feminizing treatment should be screened for breast cancer if they have been on estrogen for 30 years and are older than 50.^15^ If those women have a strong family history of breast or ovarian cancer, screening should begin earlier. Screening is done with mammography. Yearly breast exams and self-exams for transgender women without family history of breast cancer are not recommended. Breast augmentation does not increase transgender women’s risk of breast cancer, but may reduce the accuracy of mammography. Transgender women with prostate tissue should be screened with an exam for enlarged prostates, if they develop symptoms. Prostate-specific antigen is not a useful marker for transgender women on estrogen. Transgender women with prostates must be screened for prostate cancer and breast cancer. Feminizing hormones increase the risk of venous thromboembolic disease, cardiovascular disease, hypertension, and prolactinomas (which is when an adenoma, or a non-cancerous tumor, of the pituitary gland overproduces the hormone prolactin). More studies are necessary to clarify these increased risks.

Transgender males with a cervix need pelvic exams every one to three years after the age of 40.^15^ Transgender men may not have engaged in vaginal penetrative intercourse and examining the vagina, cervix, and uterus may be traumatic. It is recommended to delay those parts of the exam until a good connection is established between the patient and the examiner and the patient indicates readiness. Sedation can ease the exam and make it less painful. If the patient cannot tolerate pelvic exams, hysterectomy and oophorectomy are recommended. Transgender males over the age of 60 and taking testosterone for over five years need bone scans. If transgender males have been on testosterone over five years and they have other risks of osteoporosis, they should test at age 50. Calcium and vitamin D are recommended for transgender males due to the unknown effect of testosterone on bone density.

All transgender individuals should be screened for cardiovascular health.^15^ Cardiovascular risk factors should be decreased to a minimum before masculinizing or feminizing hormonal therapies. If a patient is at a high risk for cardiovascular disease, a stress test is indicated before treatment and when hormonal treatment is initiated, the patient should begin a low dose aspirin regimen. Patients should be screened for hypertension the same as the non-transgender patient population and blood pressure should be optimized to a goal of 130 mmHg systolic or less and 90 mmHg diastolic or less. Blood pressure should be monitored every one to three months after the onset of testosterone in the first year in transgender men on hormones, especially in ones with Polycystic Ovary Syndrome (PCOS). Transgender men and transgender women should have a lipid profile check annually. High cholesterol should be treated to a level of 3.5 mmol/L in patients with no risk factors and 2.5 mmol/L in those with additional cardiac risk factors. Patients taking estrogen have a higher risk of type 2 diabetes if a family history of diabetes occurs or a weight gain of over 11 pounds. Transgender women should be screened annually with fasting blood sugar. For transgender men, screening is indicated with history of PCOS, otherwise diabetes screening guidelines are the same as those of the general population.

All transgender individuals with risk behaviors for sexual transmitted infections or blood transmitted infections (unprotected penile-vaginal or penile-anal intercourse, sharing needles) should be screened for HIV and Hepatitis B and C every six months.^15^ For transgender patients without those risk behaviors, screening once in a lifetime is recommended. All transgender patients need Hepatitis B vaccinations. All transgender patients should be screened for depression (e.g., Patient Health Questionnaire) and, if the screen is positive, referred to a transgender competent mental health provider.

## Trends, Movements, and Expectations

Despite the growing evidence supporting the value of treating transgender patients in a mindful, supportive setting, the transgender health curriculum in most medical provider curricula remains unchanged. The AAMC has incorporated transgender educational material on MedEdPORTAL (https://www.mededportal.org/collections) with over 100 articles, presentations, and tools. Hospitals lack transgender policies and the medical environment remains a haunting experience for many transgender individuals. Health care provider attitudes toward transgender patients show bias and lack of perceived knowledge to treat them.

The transgender health movement is young, yet showing positive changes in health care learners when proper exposure and education take place.^22^ Although there remains a lack of medical education programs for transgender health, research supports introducing this topic early during clinical education of clinicians.^13^ Until transgender health care and cultural competence are mandated in medical curricula, the multiple significant obstacles remain facts in the health of over one million transgender people.^5^
